# Detection of treponemes in digital dermatitis lesions of captive European bison (*Bison bonasus*)

**DOI:** 10.1371/journal.pone.0255921

**Published:** 2021-08-09

**Authors:** Stefan Hoby, Tim K. Jensen, Isabelle Brodard, Corinne Gurtner, Richard Eicher, Adrian Steiner, Peter Kuhnert, Maher Alsaaod

**Affiliations:** 1 Berne Animal Park, Bern, Switzerland; 2 Center for Diagnostic, Technical University of Denmark, Kongens Lyngby, Denmark; 3 Institute of Veterinary Bacteriology, Vetsuisse Faculty, University of Bern, Bern, Switzerland; 4 Institute of Animal Pathology, Vetsuisse Faculty, University of Bern, Bern, Switzerland; 5 Clinic for Ruminants, Vetsuisse Faculty, University of Bern, Bern, Switzerland; University of Lincoln, UNITED KINGDOM

## Abstract

A newly-discovered foot disease of unknown origin in captive European Bison (*Bison bonasus*) was recently detected at Berne Animal Park. Dermatitis of the interdigital cleft of varying degrees of severity was diagnosed in all animals (*n* = 10). The aim of this study was to describe the gross and histological lesions of the interdigital cleft found in 10 captive European bison and to identify involved potential pathogens in affected feet using molecular-based methods for *Treponema* spp., *Dichelobacter nodosus* and *Fusobacterium necrophorum*. Lesions were scored according to the degree of gross pathology at limb level. In a single animal, the gross lesions were restricted to focal lesions on the dorsal aspect of the digital skin of each foot (score 1), whereas all other animals showed at least one foot with extended lesions including the interdigital cleft (score 2). The presence of viable spirochaetes was observed in all animals using dark field microscopy. Applying fluorescence in situ hybridisation (FISH) on biopsies, *Treponema* spp. were identified, infiltrating the skin lesions in varying numbers in nine animals. Nested PCRs for *Treponema medium*, *Treponema phagedenis* and *Treponema pedis* of swab samples showed three positive animals out of ten for the latter two, whereas pooled biopsy samples were positive in all ten animals for at least *T*. *phagedenis* (9/10) and/or *T*. *pedis* (7/10), while all samples were negative for *T*. *medium*. However, none of these *Treponema* species could be isolated and sequence analysis of the amplified products showed 100% match of 365 base pairs (bp) to *Treponema* phylotype PT3 and almost full match (530 of 532 bp, 99.6%) to *Treponema* phylotype PT13. The presence of *T*. *phagedenis*, PT3 and PT13 phylotypes was confirmed by FISH analyses. The phylotypes of *T*. *phagedenis* were present in all hybridized positive biopsies of *Treponema* spp., and PT13 and PT3 were less abundant. Neither *D*. *nodosus* nor *F*. *necrophorum* were detected. The histological *Treponema* score was mostly mild. Digital dermatitis in captive European Bison is contagious and differs from bovine digital dermatitis, concerning associated pathogens as well as gross appearance.

## Introduction

The European bison or wisent (*Bison bonasus*) is the largest European land mammal and has been extinct in the wild since 1927. However, European bison have since been reintroduced into Europe initiating in Poland in 1952 [1]. At present, the species is listed as near threatened according to the International Union for Conservation of Nature (IUCN) Red List, among other reasons due to its susceptibility to disease [2]. It is unclear whether the species has always shown weak resistance to disease or if immunity has declined, due to limited genetic heterogeneity [3]. Currently, the population is estimated at over 7000 individuals, with approximately 480 individuals listed in the European Endangered Species Programme. Disease susceptibility includes several bovine diseases, such as malignant catarrhal fever [4], bluetongue [5] and various respiratory tract diseases such as tuberculosis, which is still considered the most threatening health problem [6, 7]. With the exception of foot and mouth disease outbreaks [8], foot lesions have historically not been reported, and in a recent study of 234 wild individuals, pathological foot conditions were not mentioned [9].

Digital dermatitis (DD) is an infectious, inflammatory skin disease, first described in cattle [10]. It presents in other species such as sheep (contagious ovine digital dermatitis [11]), dairy goats [12], pigs (skin and tail lesions [13, 14]) and free-ranging elk (*Cervus elaphus*) [15], which suggests that cloven-hoofed animals are susceptible to DD. In dairy cattle, the disease is endemic and considered worldwide as a serious issue compromising the welfare and productivity of affected animals [16]. It is characterized by superficial erosive dermatitis as the initial stage, which progresses to ulcerative and/or proliferative lesions located mainly at the plantar aspect of the interdigital cleft or occasionally within the interdigital cleft [17].

Risk factors include moisture, poor conditions of hygiene, contact with affected animals and contaminated hoof trimming instruments [18, 19]. It is a multifactorial polymicrobial infectious disease, and most commonly observed bacteria associated with bovine DD belong to multiple species of the genus *Treponema* that are considered central in the etiology of DD [20]. Bovine DD lesions are generally associated with *Treponema medium*, *Treponema phagedenis* and *Treponema pedis* [21]. The presence of *Treponema* spp. has been shown to be associated with necrosis of affected tissue as investigated by fluorescent in situ hybridization (FISH) in biopsy specimens of bovine DD [22]. Histopathologically, DD lesions show epithelial hyperplasia with hyperkeratosis, loss of the stratum corneum and/or granulosum, necrosis of the epidermis leading to ulceration, and the *Treponema* spp. are present in deep layers of the affected tissue [23–25]. *Treponema* are gram-negative anaerobic bacteria that are very difficult to culture [26, 27]. Polymerase chain reaction (PCR)-based methods contributed to overcome the difficulties associated with isolation and cultivation of the fastidious treponemes. Despite the identification of the widening host range, there have been no reports of treponemes being implicated in foot pathologies of European bison.

This study was designed to describe the gross and histological lesions of the interdigital cleft found in captive European bison. Laboratory diagnostic tests were performed with the goal of identifying pathogens in clinically affected feet using molecular-based methods for *Treponema* spp., *Dichelobacter nodosus* (*D*. *nodosus*) and *Fusobacterium necrophorum* (*F*. *necrophorum)*.

## Materials and methods

### Ethical statement

The study protocol was performed in accordance with the Swiss animal welfare regulations and approved by the animal experimentation committee of the canton of Berne, Switzerland (BE89/18, 30672).

### Animals and housing

At Berne Animal Park, European bison are kept on a 49,000 m^2^ outdoor enclosure. The area is covered by native deciduous forest (mainly *Fagus sylvatica*, *Quercus robur*, *Acer platanoides*, *Alnus glutinosa*, *Betula pendula*, *Pinus sylvestris*) and the natural floor. The multipurpose indoor enclosure with concrete floor is equipped with movable walls to separate into eight boxes (2 m x 3.1 m) for medical and transport purposes. In front of the boxes, a fenced corral of 770 m^2^ with marlstone/sand floor is situated, which allows temporary separation of the group for cleaning of the outdoor enclosure. The group consists of four to five breeding females, a breeding male and their offspring. The latter is translocated to other institutions, reintroduction areas or slaughtered at the age of 1 to 2 years. The whole group of usually 10 to 12 individuals is kept together at all times. The enclosure is shared with a group of five to eight red deer *(Cervus elaphus)*. Diet consists of hay *ad libitum*, branches, pellets (Browser and Grazer, Granovit, Kaiseraugst, Switzerland) and beet in winter and fresh grass in summer. Salt licks (KNZ, Arnhem, Netherlands) are provided *ad libitum*.

### Animal preparation, locomotion scoring and blood sampling

Initially, random sampling was performed during diagnostic, curative or prophylactic medical treatments in the frame of routine zoo veterinary practice, as well as during captures related to transports. After detection of the first foot lesions, the whole European bison herd (*n* = 10) was investigated (S1 Dataset).

The day before sampling, locomotion was scored by the first author (SH) according to a five graded scoring system established by Sprecher et al. [28] for cattle, where 1 = non lame and 5 = severely lame based on gait attributes of back arch, short-striding and reluctance to bear weight on one or more limbs. Afterwards, individual animals were separated in a box and fasted overnight. Medetomidine (Medetomidin HCL, Christoffel Apotheke, Bern, Switzerland, 0.08 mg/kg) and ketamine (Ketamin HCL, Christoffel Apotheke, Bern, Switzerland, 2.5 mg /kg) were applied per estimated body weight (bw) in a mixed syringe per dart gun (Daninject, Gelsenkirchen, Germany) in the shoulder or thigh intramuscularly (i.m.). The animals were placed in right lateral recumbency, received 1 liter of oxygen/100 kg bw/min per nasal probe and carprofen (Rimadyl Rind, Zoetis, Switzerland) in a dosage of 1.4 mg/kg bw i.m. For reversal, atipamezole (Alzane, Gräub, Switzerland, 0.4 mg/kg bw i.m.) was administered.

Blood was drawn from the *Vena jugularis* from each animal, immediately cooled at +4 °C and after centrifugation (2000 rpm, 10 min), serum was stored at -80 °C to later investigate the trace elements cobalt, copper, iodine, manganese, selenium and zinc by mass spectrometry.

### Collection of swab and biopsy samples

After removing the dirt from the interdigital cleft with a dry paper towel, individual swab samples were collected from all four feet using sterile, dry cotton swabs. The samples were taken by rubbing the swab from the interdigital cleft to the bulb. The individual swabs were immediately placed into Eppendorf tubes containing 1 ml lysis buffer (4 M guanidine thiocyanate, 0.01 M Tris–HCl, 1% β-mercaptoethanol) and were gently stirred for 2 min. The swabs were then discarded and the remaining lysates transferred to the laboratory for DNA extraction. Thereafter, the interdigital cleft was washed with tap water to remove the organic debris attached to the skin`s surface, and then standardized photographs were taken from the feet with a digital camera and stored for visual analysis. Thereafter, the interdigital skin was disinfected with 70% ethanol, and skin specimens were collected using a sterile 6 mm punch biopsy with a maximum depth of 7 mm (Henry Schein, Lyssach, Switzerland) under sterile conditions from the centre of the lesions if present. The biopsies were removed with a # 10 sterile scalpel blade and transferred directly to a sterile Petri dish. Each biopsy was cut into three parts for molecular analysis, visualization of spirochaetes, and histopathology, respectively. Following biopsy sampling, the lesions were treated locally with salicylic acid paste (Novaderma, Streuli Tiergesundheit, Switzerland), and a loose bandage was applied.

Five red deer (*Cervus elaphus*) shared the enclosure with the European bison. They were individually clinically examined and swabbed in the same time period as the European bison [29].

### Processing of swab and biopsy samples

For molecular analysis, a third of each biopsy was immediately placed into an Eppendorf tube containing 1 ml lysis buffer for DNA extraction as previously described for cotton swabs. The four biopsies of each animal were pooled for molecular analysis.

For visualization of spirochaetes, a third of each biopsy placed in petri dishes was minced into small fragments (~ 1 mm2 cubes) using a sterile scalpel blade in an anaerobic workstation (mixed gas: 85% N2, 10% H2, 5% CO2 and 37 °C) (Whitley DG250; Meintrup DWS Laborgeräte GmbH). The minced biopsy was then transferred to 7 ml Oral Treponeme Enrichment Broth (OTEB; Anaerob Systems, Morgan Hill, CA, USA) supplemented with 10% rabbit serum (Gibco, life technologies, USA), 5 μg/ml rifampicin, 5 μg/ml enrofloxacin and incubated anaerobically at 37 °C. After 4 hours of incubation an aliquot was taken to verify the presence of viable spirochaetes using dark field microscopy at 20-40x magnification. Cultures were further processed as previously described by Brodard et al. [27] to isolate *Treponema* spp.

For histological examination, a third of each biopsy sample was fixed in 10% neutral buffered formalin, embedded in paraffin, sectioned at 4 μm, and mounted on glass slides. From all biopsy samples two sections were made and either stained with hematoxylin and eosin (H&E) or with a silver stain (Wartin Starry) to check for spirochaetes. Subsequently, the samples were evaluated by light microscopy.

For FISH analysis, serial sections of 4 μm of all formalin-fixed, paraffin embedded biopsies were cut and mounted on SuperFrost+ slides (Menzel-Gläser, Braunschweig, Germany) and hybridized as previously described [25].

The oligonucleotide probes used in this study were listed in S1 Table and included probes specific for the domain *Bacteria*, the genus *Treponema*, and 17 species/phylotypes including *D*. *nodosus*, *T*. *pedis*, *T*. *phagedenis*, *T*. *medium*, “*T*. *refringens”*, *T*. *denticola*, and *T*. *brennaborensis*, PT3 and PT13. In brief, hybridization was carried out at 45° C for 16 h and at a final probe concentration of 5 ng/μL. After hybridization, the slides were washed in washing buffer, rinsed in water, air dried, and mounted in Vectashield (Vector Laboratories Inc., Burlingame, CA, USA) for fluorescence microscopy.

The probe for domain *Bacteria* was 5’ labeled with fluorescein isothiocyanate (FITC) and all other bacteria probes were 5’ labeled with the isothiocyanate derivative Cy3 (Eurofins MWG Operon, Ebersberg, Germany). The probe for domain *Bacteria* was used on all slides in combination with one other probe for individual bacterial species. Control samples for FISH were prepared by injecting pure cultures of the following bacterial species suspended in a 0.9% sterile saline solution into sterile porcine lung samples: *D*. *nodosus* (CCUG 27824) and *T*. *phagedenis* (ATCC 27087).

An Axioimager M1 microscope (Carl Zeiss, Oberkochen, Germany), equipped for epifluorescence with a 100-W HBO lamp and filter sets 24 (excitation at 485/578 nm), 38 (excitation at 470 nm), and 43 (excitation at 550 nm) for the detection of double staining (FITC and Cy3 or AF555) and single staining (FITC, Cy3, AF555), respectively, was used to examine the hybridized specimens. Images were obtained using an AxioCam MRm version 3 FireWire monochrome camera and the AxioVision software, version 4.5 (Carl Zeiss).

### Classification of gross lesions

After blinding the photographs, lesions were scored according to the degree of gross pathology at limb level by the first and last authors as (0) no lesions; (1) focal lesion on the dorsal aspect of the digital skin; and (2) extended lesion involving the dorsal digital skin and the interdigital cleft. Furthermore, hair growth around the lesion was classified as (0) normal or (1) irregular.

### DNA extraction, PCR assays and sequencing

Extraction of DNA from lysis buffer samples was done as described previously by Stauble et al. [30] using a semi-automatic extraction robot (King Fisher Duo Prime, Thermo Fisher Scientific, Reinach, Switzerland). The Quantus^™^ Fluorometer (Promega, Madison, USA) was used to quantify DNA. Purified DNA was stored at -20 °C until further processing.

Specific nested PCR assays for *T*. *medium*, *T*. *phagedenis*, and *T*. *pedis*, were performed on swab and pooled biopsy samples according to Evans et al. [31] using FIREPol^®^ Master Mix Ready to load with 12.5 mM MgCl_2_ (Solis Biodyne, Tartu, Estonia). Genomic DNA from the types strains was used for positive controls (*T*. *medium* ATCC 700293^T^, *T*. *phagedenis* B43.1^T^, *T*. *pedis* DSM 18691^T^). From seven animals (No. 2, 4, 5, 6, 8, 9, and 10, Table 1), the PCR products were purified with the ‘High pure PCR purification Kit’ (Roche Diagnostics, Rotkreuz, Switzerland). Sanger sequencing was performed at Microsynth, Balgach, Switzerland using corresponding PCR primers. Sequences were aligned and edited using Sequencher (Gene Codes, Ann Arbor, MI, USA) followed by sequence comparison against GenBank using BLAST (www.ncbi.nlm.nih.gov).

**Table 1:** Clinical scores, hair scores, *Treponema* visualization, PCR results, histopathological evaluation and fluorescent in situ hybridization results of biopsies obtained from 10 European bison (*Bison bonasus*) at animal and limb level.

Animal No. (^1^sex, age in months)	^2^Limb	^3^Clinical score (0–2)	^4^Hair score (0–1)	^5^Viable *Treponema*	^6^Species-specific *Treponema* nested PCR		^7^*D*. *nodosus* competitive rtPCR	^8^*F*. *necrophorum* rtPCR	Histopathological evaluation		^11^ Fluorescent in situ hybridization				
					Swab	Biopsy	Swab and biopsy	Biopsy	^9^ Histo-pathology score (keratino-lysis score / dermatitis score) (0–3)	^10^Amount of spirochaetes in Warthin -Starry stain (0–3)	*Treponema* spp. score (0–3)	PT13 (0–1)	*T*. *phagedenis* (0–1)	PT3 (0–1)	*D*. *nodosus* score (0–1)
1 (f, 19)	FL	2	1	Yes	*T*. *phagedenis*	*T*. *pedis* *T*. *phagedenis*	Negative	Negative	0 / 1	1	1	1	1	1	0
	FR	2	1		*T*. *pedis* *T*. *phagedenis*				0 / 3	1	1	1	1	0	0
	RL	2	1		Negative				0 / 1	1	1	1	1	0	0
	RR	2	1		Negative				0 / 2	0	1	1	1	1	0
2 (m, 9)*	FL	2	1	Yes	Negative	*T*. *pedis* *T*. *phagedenis*	Negative	Negative	1 / 1	1	0	-^§^	-	-	0
	FR	1	1		Negative				1 / 1	1	1	1	1	0	0
	RL	2	1		Negative				1 / 1	1	1	1	1	1	0
	RR	2	1		Negative				1 / 2	0	1	0	1	1	0
3 (f, 45)	FL	2	1	Yes	Negative	*T*. *phagedenis*	Negative	Negative	0 / 1	0	0	-	-	-	0
	FR	2	na^12^		Negative				1 / 1	1	0	-	-	-	0
	RL	2	1		Negative				0 / 1	1	1	1	1	0	0
	RR	2	1		Negative				1 / 2	0	1	1	1	1	0
4 (m, 10)*	FL	2	1	Yes	Negative	*T*. *pedis* *T*. *phagedenis*	Negative	Negative	1 / 1	1	2	0	1	0	0
	FR	2	1		Negative				1 / 1	1	2	1	1	0	0
	RL	2	1		Negative				0 / 2	2	1	1	1	1	0
	RR	2	1		Negative				0 / 2	2	1	1	1	1	0
5 (f, 175)*	FL	2	1	Yes	Negative	*T*. *phagedenis*	Negative	Negative	1 / 3	1	0	-	-	-	0
	FR	2	0		Negative				0 / 1	0	0	-	-	-	0
	RL	2	1		Negative				1 / 2	1	2	1	1	1	0
	RR	2	1		Negative				0 / 2	1	0	-	-	-	0
6 (f, 154)*	FL	2	0	Yes	Negative	*T*. *phagedenis*	Negative	Negative	1 / 1	0	0	-	-	-	0
	FR	2	0		Negative				0 / 0	0	0	-	-	-	0
	RL	2	1		Negative				1 / 1	0	0	-	-	-	0
	RR	2	0		Negative				1 / 1	0	0	-	-	-	0
7 (m, 9)	FL	1	0	Yes	Negative	*T*. *pedis* *T*. *phagedenis*	Negative	na^12^	0 / 0	1	0	-	-	-	0
	FR	1	0		Negative				1 / 0	2	0	-	-	-	0
	RL	1	1		Negative				3 / 3	2	3	1	1	1	0
	RR	1	0		Negative				0 / 1	2	0	-	-	-	0
8 (f, 167)*	FL	1	1	Yes	Negative	*T*. *pedis* *T*. *phagedenis*	Negative	Negative	0 / 0	0	0	-	-	-	0
	FR	2	1		Negative				0 / 1	0	0	-	-	-	0
	RL	2	1		Negative				0 / 1	0	0	-	-	-	0
	RR	2	1		Negative				2 / 2	1	1	0	1	0	0
9 (m, 168)*	FL	1	0	Yes	Negative	*T*. *pedis*	Negative	Negative	0 / 1	0	0	-	-	-	0
	FR	1	1		Negative				0 / 1	0	0	-	-	-	0
	RL	2	1		*T*. *pedis* *T*. *phagedenis*				0 / 1	1	2	1	1	0	0
	RR	1	1		Negative				0 / 1	0	0	-	-	-	0
10 (f, 169)*	FL	2	0	Yes	Negative	*T*. *pedis* *T*. *phagedenis*	Negative	Negative	0 / 1	0	0	-	-	-	0
	FR	2	0		*T*. *phagedenis*				0 / 1	0	1	0	1	0	0
	RL	2	0		*T*. *pedis*				0 / 1	0	0	-	-	-	0
	RR	2	0		Negative				1 / 2	0	0	-	-	-	0

^1^ sex: f = female, m = male

^2^ Foot: FL = fore left, FR = fore right, RL = rear left, RR = rear right

^3^ Lesions were scored as (0) no lesions; (1) focal lesion on the dorsal aspect of the digital skin; and (2) extended lesion involving the dorsal digital skin and the interdigital cleft.

^4^ hair growth around the lesion was classified as (0) normal or (1) irregular growth.

^5^ The presence of viable motile spirochaetes on animal level based on morphological features as described by Radolf [35] by using dark field microscopy

^6^ Species-specific nested PCR assays as described by Evans et al. [31], to identify the presence of specific treponeme phylogroups (*T*.*pedis*, *T*.*phagedenis*, and *T*.*medium*). Swabs were tested on foot level, biopsies were tested on animal level after pooling the four samples.

^7^ The presence of *D*. *nodosus*-specific DNA as evaluated by a real-time PCR for *aprV2* and *aprB2* according to Stauble et al. [32]

^8^ The presence of *F*. *necrophorum* DNA as evaluated by a real-time PCR according to Jensen et al. [34]

^9^ Scoring according to Read and Walker [23] and modified by Klitgaard et al. [24]. Degree of keratinolysis: 0 = no changes; 1 = focal; 2 = moderate; 3 = extensive. Degree of chronic lymphoplasmacytic perivascular dermatitis: 0 = absent; 1 = mild; 2 = moderate; 3 = severe.

^10^ The number of spirochaetes in the Warthin-Starry stain: 0 = none visible; 1 = minimal amount; 2 = moderate amount; 3 = high amount.

^11^ Fluorescent in situ hybridization for the detection of *Treponema* spp.: 0 = no hybridization, 1 = sparse hybridization, 2 = moderate hybridization, and 3 = strong hybridization. *D*. *nodosus*, PT13, *T*. *phagedenis*, PT3: 0 = no hybridization; 1 = positive hybridization.

^12^ na = not available

* From these animals, PCR products were available for sequence analysis.

^§^ Not performed as the biopsy was negative for *Treponema* spp (no hybridization)

Detection of virulent (*aprV2-*positive) and benign (*aprB2*-positive) *D*. *nodosus* was done with the competitive real-time PCR as described in detail by Stauble et al. [32] using the TaqMan^®^ Fast Advanced Master Mix (Thermo Fisher Scientific) and adapted by Kuhnert et al. [33].

Detection of *Fusobacterium necrophorum* was done by real-time PCR as described by Jensen et al. [34].

### Histopathological evaluation

The samples were classified according to the scoring developed by Read and Walker [23] and modified by Klitgaard et al. [24]. The scoring is based on histological changes in the epidermis and dermis. Characteristic changes of DD are as follows: i) a focal circumscribed acanthotic epidermis with or without parakeratotic papillomatous proliferation, ii) the loss of the stratum granulosum, or iii) the presence of an inflammatory infiltrate in the dermis. The histopathologic scoring of the lesions was performed considering the degree of keratinolysis in the stratum corneum as well as the degree of the chronic, perivascular and lymphoplasmacytic dermatitis present. Keratinolysis is defined as the damage to the corneocytes of the stratum corneum, visible as intracellular edema, degeneration and lysis of the corneocytes. Degree of keratinolysis: 0 = none present; 1 = focal; 2 = moderate; 3 = extensive. Degree of chronic dermatitis: 0 = no changes present; 1 = mild, chronic, perivascular, lymphoplasmacytic, dermal inflammation; 2 = moderate, chronic, perivascular, inflammation. 3 = severe, chronic, perivascular, lymphoplasmacytic inflammation. Furthermore, the number of spirochaetes visible in the Warthin-Starry stain on top of and in the epidermis was classified: 0 = none visible; 1 = minimal amount; 2 = moderate amount; 3 = high amount.

### FISH scoring

For each of the applied *Treponema* probes the hybridization signal was scored from 0 to 3 according to Klitgaard et al. [24]: 0 = no hybridization, 1 = sparse hybridization, 2 = moderate hybridization, and 3 = strong hybridization. The presence of *D*. *nodosus* was scored as 0 = no hybridization, or 1 = positive hybridization.

## Results

**Fig 1:**
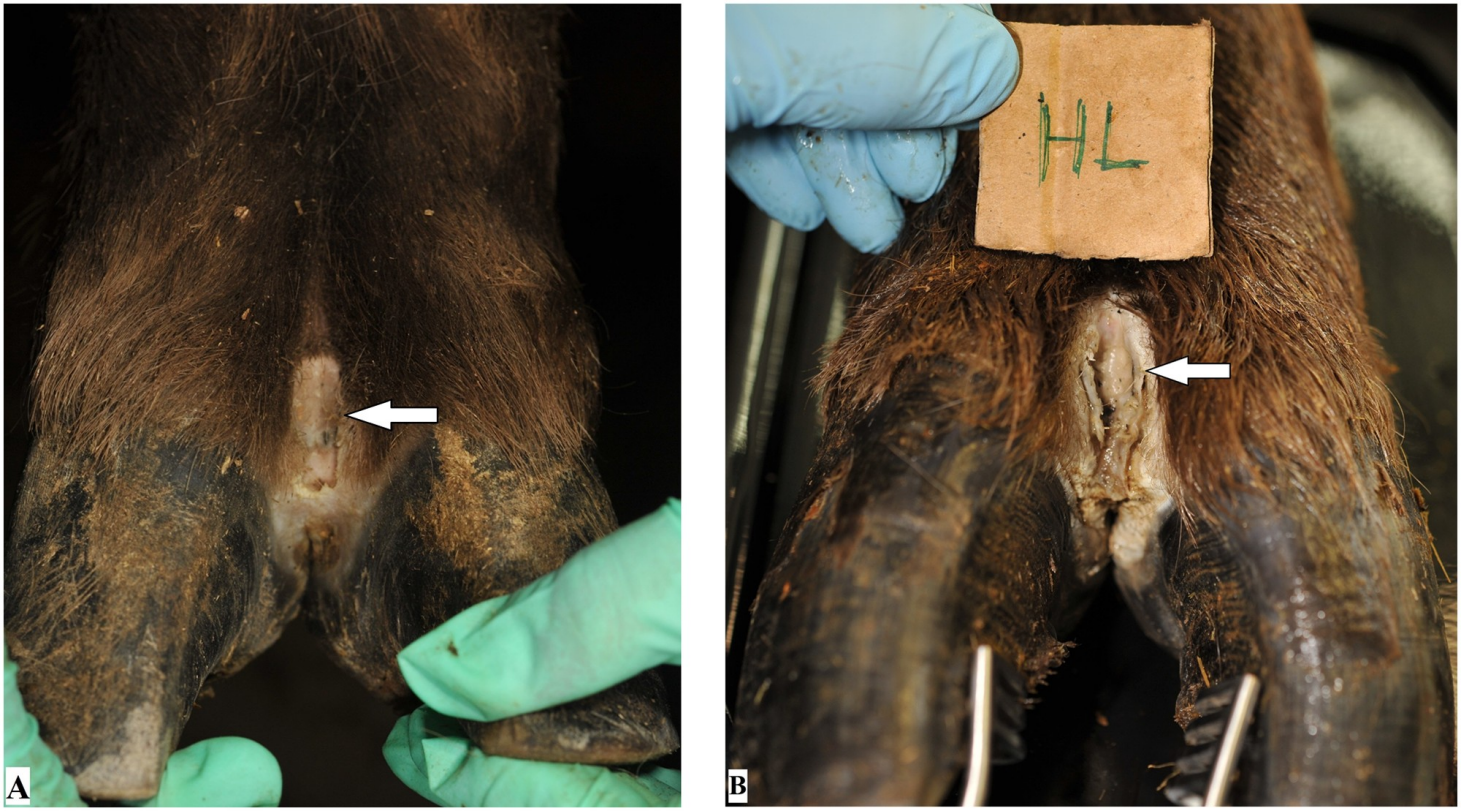
Feet of captive European bison (*Bison bonasus*) with digital dermatitis lesions (indicated with arrows). (A) Left front foot of animal no. 7 with a focal lesion (score 1) on the dorsal aspect of the digital skin. (B) Left hind foot of animal no. 10 with an extended lesion involving the dorsal digital skin and the interdigital cleft (score 2) of the digital skin. Hair growth around the lesions in A and B was rated normal (score 0).

Macroscopically, all animals showed digital lesions on all feet. Demographic data and results on animal and foot level are presented in Table 1. In a single animal, they were restricted to focal, well-demarcated, pale, eroded to ulcerated areas on the dorsal aspect of the digital skin of each foot (score 1). All other animals showed at least one foot with extended lesions involving the interdigital cleft (score 2) (Fig 1). These were well-demarcated, malodorous, ulcerated lesions, sometimes circumscribed by pale fibrous tissue, consistent with second intention wound healing, interpreted as granulation tissue. Hair growth was classified normal on all feet of one individual (score 0), and all other animals showed at least one foot with irregular hair growth (score 1). One single foot could not be scored due to poor photograph quality. In the clinical exam, a single animal (No. 10, Table 1) showed a bilateral forelimb lameness (score 3), and all the other animals were scored non lame (score 1). The feet of red deer were clinically healthy and swab samples tested negative for *Treponema* spp. and *D*. *nodosus*.

Trace elements of cobalt (mean 0.59 micrograms/l, min. 0.36- max. 0.86), copper (844 mcg/l (670–930)), selenium (86 mcg/l (60–180)) and zinc (890 mcg/l (540–1080)) were rated normal, iodine (167 mcg/l (91–340)) elevated and manganese (1.62 mcg/l (1.1–2.8)) decreased when using cattle values as reference.

### Bacteriological evaluation

The presence of viable spirochaetes was observed in all animals using dark field microscopy based on morphological features (helically coiled, corkscrew-shaped organisms, 6 to 15 μm long and 0.1 to 0.2 μm wide with rapid rotation around its longitudinal axis) as described by Radolf [35]. It was not possible to isolate any *Treponema* spp. despite successfully doing so in our laboratory from bovine DD samples [27].

#### PCR detection of *Treponema* species, D. nodosus and F. necrophorum and sequencing

The results of *Treponema* species PCR assays of all 10 animals are shown in Table 1. Swab samples were positive for three out of 10 animals for both DD-associated *Treponema* species (*T*. *phagedenis* and *T*. *pedis*) and at the foot level only five out of 40 swabs were positive for *T*. *phagedenis* and/or *T*. *pedis*. In contrast, by analysing the 10 pooled biopsy samples, all animals were positive for at least one or more of the DD-associated *Treponema* species (*T*. *phagedenis* (9/10) and/or *T*. *pedis* (7/10). None of the swab and biopsy samples were positive for *T*. *medium*.

**Fig 2:**
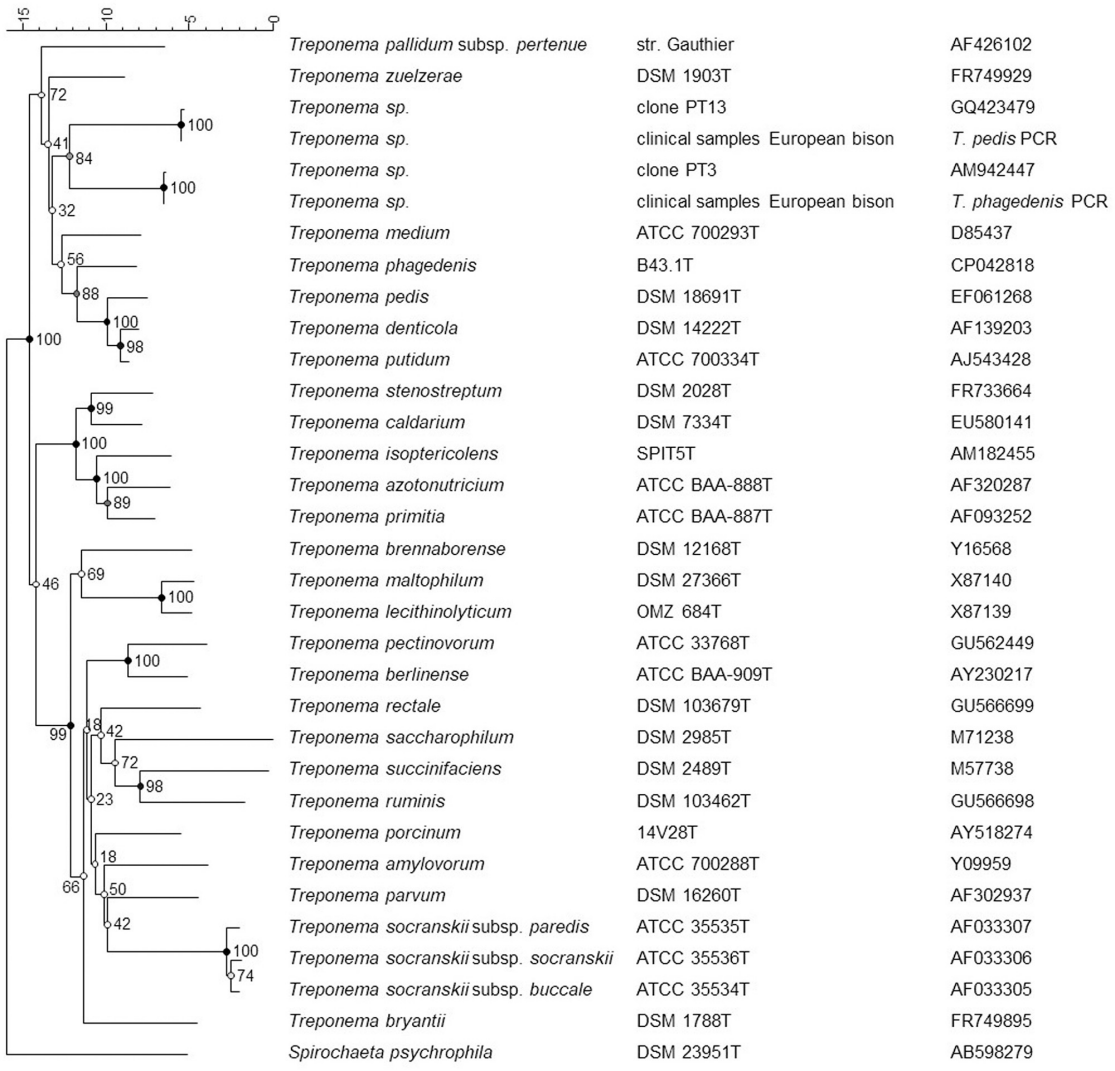
Phylogenetic tree based on partial 16S rRNA gene sequences of currently recognized species of the genus *Treponema*, the PCR products from clinical samples from lesions of seven European bison (*Bison bonasus*) and the corresponding sequences from GenBank entries of clones PT3 and PT13. *Spirochaeta psychrophila* was included as an outgroup for rooting the tree that was built in Bionumerics 7.6.3 by using Jukes-Cantor correction and Neighbour joining for cluster analysis. Bootstrap values from 500 iterations are given at branches. The bar represents percent sequence divergence. The species name, strain designation and accession number, where available, is given.

Since we were not able to isolate *T*. *phagedenis* nor *T*. *pedis*, still available pooled biopsies from seven animals (No. 2, 4, 5, 6, 8, 9, and 10) were analysed in more detail by sequencing the corresponding PCR products. The amplicons from the *T*. *phagedenis* nested PCR showed identical sequences in all cases and only showed 92% similarity to this species, but 100% match of the 365 base pairs (bp) to *Treponema* phylotype PT3 (GenBank accession number AM942447, Klitgaard et al. [24]) (Fig 2). The amplicons obtained from the *T*. *pedis* nested PCR presented identical sequences in all cases as well, but showed almost full match (530/532 bp, 99.6%) to *Treponema* phylotype PT13 (GenBank accession number GQ423479, Rasmussen et al. [25]), 95% to *T*. *phagedenis* and only 88% to *T*. *pedis* (Fig 2).

The *aprB2/ aprV2* gene of *D*. *nodosus* was not detected in any of the samples investigated, neither in the swab nor biopsy samples. All biopsy samples were PCR-negative for both *F*. *necrophorum* subsp. *necrophorum* and *F*. *necrophorum* subsp. *funduliforme*.

#### Histopathological evaluation

Table 1 shows an overview of the different scores for keratinolysis, dermatitis and the number of spirochaetes. All biopsies showed epidermal hyperplasia with formation of rete ridges covered by a thick orthokeratotic hyperkeratotic stratum corneum except for the biopsies from one foot in animals No. 6, 7 and 8. In one animal (No. 1) one biopsy revealed a parakeratotic hyperkeratosis of the stratum corneum. All animals except animals No. 9 and 10 showed erosions or ulcerations of the epidermis on at least one foot. If keratinolysis was present, it was usually focal, except for one foot in animals No. 8 (multifocal) and No. 7 (extensive). All examined animals showed changes consistent with a mild, chronic perivascular dermatitis composed of lymphocytes and plasma cells on one or more feet. In addition, seven animals showed moderate dermatitis, and two individuals showed severe dermatitis in at least one foot. Only a small to moderate number of spirochaetes was visible as black, slender longish rods in 8/10 animals and 21/40 feet in the Warthin-Starry stain. The detected spirochaetes were mainly located in the stratum corneum, or if eroded in the stratum granulosum. Only in rare occasions very few spirochaetes were found located within the stratum basale of the epidermis such as in the biopsies of the left hind limbs from animals No. 4 and 5.

#### FISH

**Fig 3:**
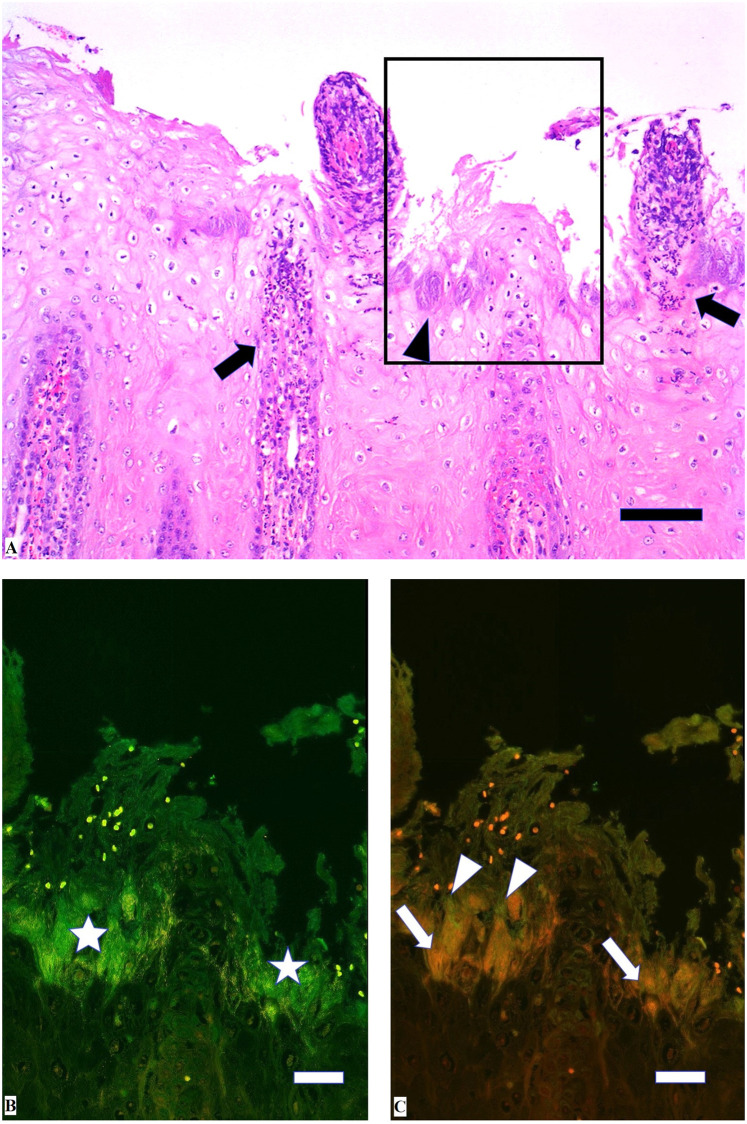
Histological appearance of interdigital skin of European bison (*Bison bonasus*) with digital dermatitis. Left hind foot of animal no. 7. A: The dermatitis is characterized by epithelial acanthosis, ballooning degeneration of keratinocytes and elongated, dermal papillae containing mixed cellular infiltrate (arrows) protruding to the surface. Large amounts of basophilic bacteria are infiltrating the superficial epidermis (arrowhead). H&E, bar 100 μm. Rectangle shows selected area for in situ hybridization on a parallel section. B: Bacteria visualized as bright green organisms (*) infiltrating the superficial epidermis. Fluorescent in situ hybridization. Bar 25 μm. C: *Treponema* organisms (arrows) infiltrating deep into the epidermis whereas other bacteria (arrowheads) are seen superficially. Double fluorescent in situ hybridization with probe for genus *Treponema* (Cy3 labelled) and for domain *Bacterium* (fluorescein labelled). Bar 25 μm.

**Fig 4:**
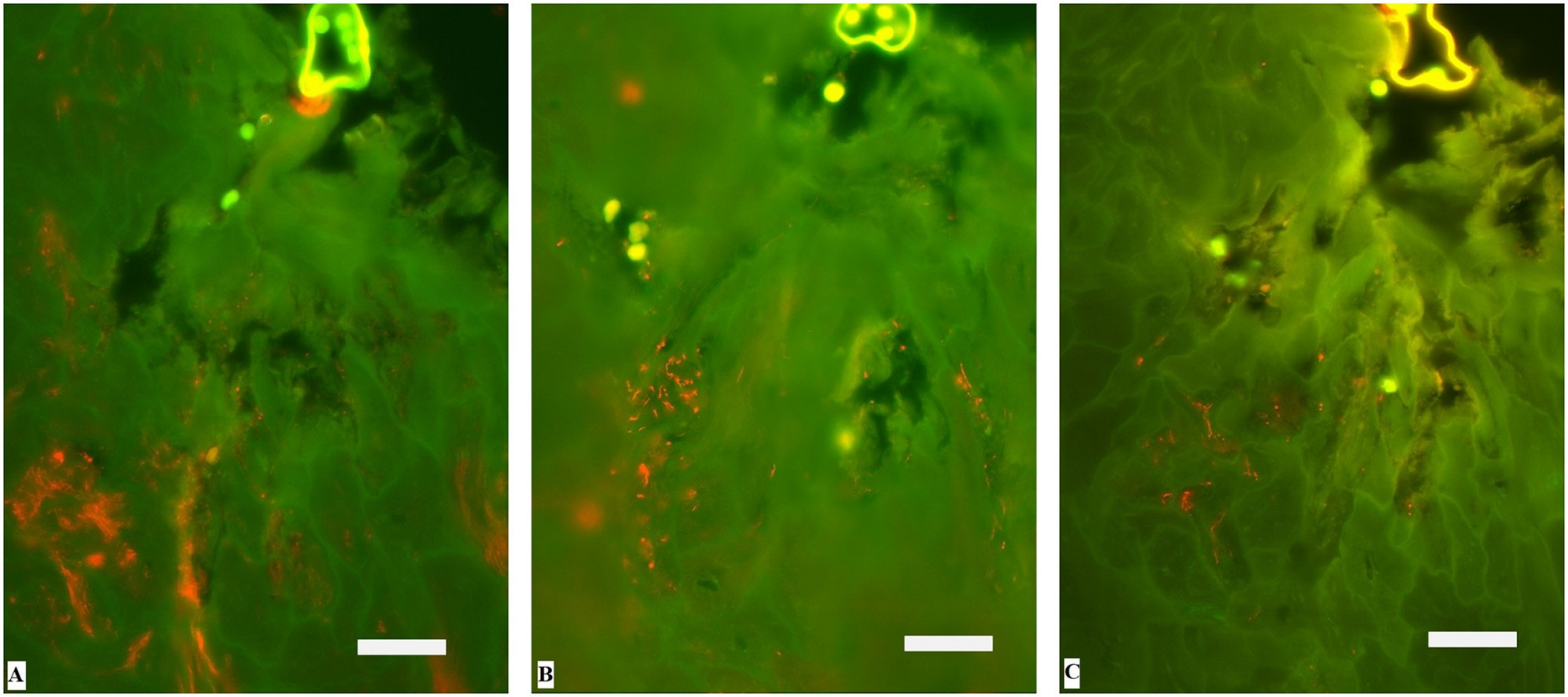
Identification of *Treponema* organisms infiltrating the interdigital skin of European bison (*Bison bonasus*) with digital dermatitis by fluorescent in situ hybridization. Three serial sections of the biopsy from the left hind foot of animal no. 5 hybridized with (Cy3 labelled) species specific oligonucleotide probes for A: *Treponema phagedenis*, B: Phylotype 13 (PT13), and C: Phylotype 3 (PT3), respectively. The *Treponema* organisms appear orange. Bar 25 μm.

Applying double in situ hybridisation with the general oligonucleotide probe for the bacterial domain (eub-338) and the probe for *Treponema* spp., treponemes infiltrating the epidermis were identified in 18 biopsies from nine animals (Table 1). The treponemes revealed a clear specific signal and were found focally within the upper most superficial layers of the epidermis with or without other bacteria with the exception of a few biopsies. Severe, extensive treponemal epidermal infiltration (score 3) that constituted more than 90% of the total bacterial population was observed in the biopsy of the left hind foot of animal no. 7. In the middle and deeper parts of the lesion, bacteria other than treponemes were negligible (Fig 3). Five biopsies displayed focal, moderate, superficial infiltration of treponemes (score 2) in ballooned degenerative epithelial cells. Additional hybridization of the *Treponema* spp. positive biopsies with the oligonucleotide probes specific for *T*. *phagedenis*, PT3 and PT13 revealed mixed, intermingled infection in 15 biopsies. *T*. *phagedenis* phylotypes were found in all *Treponema* spp. positive biopsies. Eight biopsies were positive for all three phylotypes (Fig 4), six biopsies were positive for *T*. *phagedenis* and PT13, and one biopsy was positive for *T*. *phagedenis* and PT3 and three biopsies were positive for *T*. *phagedenis* only (Table 1). Biopsies with mixed infection had highest abundance of *T*. *phagedenis* organisms and with PT3 as the less prevalent phylotype. Hybridization with the 14 other different species/phylotype specific *Treponema* probes was negative in all *Treponema* spp. positive FISH biopsies. *D*. *nodosus* was not detected by FISH in any of the formalin-fixed biopsies.

## Discussion

This is the first study reporting the detection of *Treponema* spp. in association with digital dermatitis of European bison. Our findings confirm the fact that *Treponema* spp. can infect a wide variety of hosts, ranging from wild and domestic animals to humans including now European bison, causing different macroscopical manifestations.

In European bison, foot lesions were located interdigitally in all feet, varied macroscopically from focal to extended lesions and affected both fore and hind limbs concurrently. They showed a distinct and different macroscopical manifestation in comparison with bovine DD [10], contagious ovine DD (CODD) in sheep [36] and DD in wild North American Elk (*Cervus elaphus*) [15, 37]. Typically, the initial bovine DD lesions are characterised by ulceration and formation of granulation tissue of the heel area of the hind limbs, while proliferative and hyperkeratotic lesions have been found in severe and chronic phases of bovine DD [38]. More rarely, lesions in cattle have been found in the fore limbs, in the interdigital skin or in the area of the coronary band. In small ruminants and wild elk, macroscopical lesions typically appear as dermatitis along the coronary band, and may develop into complete hoof avulsion in severe cases [39, 40].

Lameness, walking on toes and clubbing of feet are the most common clinical signs of erosive and papillomatous forms of bovine DD [23], although not all the cases of DD cause obvious clinical signs. However, only a single European bison showed a chronic, bilateral foreleg lameness (score 3/5), and all the other animals were scored non lame (score 1). This specific 14-years old cow was later slaughtered due to welfare reasons. Apart from the chronic interdigital dermatitis, this animal suffered from focal-extensive osseous metaplasia of the deep digital flexor tendons at the pedal bone insertion of the lateral claws of both forelimbs. Determining which lesion arose first and was responsible for the lameness was not possible due to chronicity, but the emergence of these lesions was likely concomitant.

Trace elements are important for the immune defence and are involved in wound healing, and were therefore included the analysis in this study. No such blood reference values have been published for European bison. However, copper, selenium and zinc were rated normal, iodine elevated and manganese lowered when compared to cattle values. In a recent study, numerous trace elements have been assessed in livers of 30 captive and free-living European bison [41]. Interestingly, deficiency of Mn was also present in 20% of captive and 37% of free-ranging animals.

Several studies have identified a strong association of specific treponeme phylogroups with bovine DD lesions [42, 43]. Accordingly, FISH studies reported the implication of specific treponeme phylogroups as the considered etiological agents of DD [24, 25]. In this study, the presence of treponemes in FISH analyses revealed that these species are implicated in the European bison foot disease. Similarly to DD in cattle and CODD in sheep [24, 31, 40], the lesions from European bison feet were polytreponemal with *Treponema* phylotypes belonging to *T*. *phagedenis*, PT3 and PT13.

The nested PCR for *Treponema* species was negative for *T*. *medium* in all analysed samples, while bands of the correct size were obtained for *T*. *phagedenis* and/or *T*. *pedis* in all pooled biopsy samples from all animals. Sequence analysis of the PCR products of seven animals then showed that these were not species-specific. In fact, they matched sequences derived from yet uncultured *Treponema* phylotypes PT3 and PT13. This indicates, that *Treponema* species other than *T*. *phagedenis* and *T*. *pedis* are present in the feet of European bison and that the nested PCR is not specific enough for this host. This is due to the fact, that the 16S rRNA gene used as target is highly conserved and present in all bacteria. Yet unknown or, in this case, yet uncultured *Treponema* species can therefore result in PCR amplification if the primers used are able to bind in the corresponding region, which is especially the case in the absence of the original target sequence. Thus further PCR diagnostic systems are needed, which are more species-specific, ideally targeting species-specific genes.

FISH analyses revealed a high abundance of *T*. *phagedenis* organisms and PT3 and PT13 as the less prevalent phylotypes. *T*. *phagedenis*, PT3 and PT13 have previously been associated with bovine DD. *T*. *phagedenis* was amongst the most frequent and abundant types, which was also found in deeper parts of the bovine DD lesions by FISH [22, 44]. PT3 belongs to the “*T*. *refringens"* cluster and was amongst the most frequently detected phylotypes in bovine DD lesions (85% of the samples) and represented 100% of the invasive bacteria identified and might have been present at an earlier state of infection [24]. PT13 was 94% identical to "*T*. *refringens"* [25], and a previous study investigated the prevalence of PT13 and PT3 (38.2% and 30.4%, respectively) from DD lesions of grazing cattle using FISH [44].

The results of the competitive real-time PCR for both, the benign (*aprB2*-positive) and virulent (*aprV2*-positive) strains of *D*. *nodosus*, additionally to FISH analysis, were negative in all investigated samples [29]. Furthermore, all tested samples were PCR-negative for *F*. *necrophorum*. These findings are in contrast to the condition interdigital dermatitis (IDD) that affects cattle [45]. The histopathology of IDD is similar to DD, and *D*. *nodosus* and *F*. *necrorphorum* are commonly associated with IDD in cattle [45, 46]. Lesions may be proliverative, erosive and develop to papillomatous lesions associated with treponemes [47]. *D*. *nodosus* is the main pathogen involved in the multifactorial disease of ovine foot rot [48]. Sheep are primary hosts for both virulent and benign *D*. *nodosus*, while cattle carry benign *D*. *nodosus* [49].

In our study, only one foot scored as DD score 3 in FISH and histology (strong hybridisation in FISH and severe dermatitis). In this case, *Treponema* spp. infiltrated deep into the epidermis, whereas other bacteria were located superficially. Similarly, massive and deep colonization of treponemes was found in deep parts of lesions with severe epidermal damage (score 3) at the border of healthy and affected tissue using FISH in bovine DD [44]. Moreover, studies on bovine DD have reported that multiple *Treponema* spp. are not only consistently found in the superficial layer of the epidermis, but also in the deep layer invading the dermis [24, 50].

The DD found in European bison of this study was contagious, as all herd members of different sex and age were affected. However, infection and transmission routes of *Treponema* species in European bison are not known, and the spread throughout the herd could not be assessed in this cross-sectional study. The feet of red deer that share the enclosure with the European bison at Berne Animal Park were clinically healthy [29], and swab samples tested negative for *Treponema*, suggesting that this species is not affected or acts as a spill over species for European bison *Treponema*. Bovine treponemes are assumed to be transmitted via environmental (e.g., manure, slurry) and direct skin contact between affected animals [51–53]. An epidemiological study including further European bison collections is required to elucidate the prevalence, infection reservoirs and transmission routes for future monitoring and management of this disease. This is especially important in light of increasing densities of free-living European bison that may favour transmission of infectious disease [54].

## Conclusion

This study reveals an expanding host range for *Treponema* spp. associated digital dermatitis to European bison. The sequencing results suggest *Treponema* species related to phylotypes PT3 and PT13 are involved in the pathogenesis of digital lesions of European bison. Additionally, the presence of *T*. *phagedenis*, PT3 and PT13 phylotypes was confirmed by FISH analyses. We encourage continued vigilance and surveillance of DD in European bison and further research to better characterize the disease.

## Supporting information

S1 Dataset Animals and their findings included in this study. (XLSX)

S1 Table The oligonucleotide probes used in this study. (DOCX)
