# Supplementary material for: Detection of treponemes in digital dermatitis lesions of captive European bison (Bison bonasus)
Source: PLoS One. 2021 Aug 9;16(8):e0255921. doi: 10.1371/journal.pone.0255921 (PMC8352065; doi:10.1371/journal.pone.0255921)
Supplement: S1 Table — (DOCX) [file pone.0255921.s002.docx]

**S1 Table.** Names and sequences of 16S rRNA-targeting oligonucleotide probes used in European bison (*Bison bonasus*).

| **Phylotype /**  **Species** | **Oligonucleotide probes** | **Sequence** | **Reference** |
| --- | --- | --- | --- |
| Bacteria | S-D-eub-338 | 5´-GTC ATT CCA TCG AAA CAT A-3´ | (2) |
| *Treponema* spp. | S-S-TrepGenus-725 | 5´-CAG AAA CYC GCC TTC GCC-3´ | (3) |
| PT1 | S-S-Trep-Dig3-432 | 5´-CAT CCC AGT ATC ATT CCC-3´ | (3) |
| PT2 | PT2 | 5'-C CGT CAT CTT TGC ATC ATT ACC-3' | (4) |
| PT3 | S-S-Trep-Dig4-432 | 5´-CAT CTC AGT GTC ATT CCC-3´ | (3) |
| PT4 | S-S-Trep-DDKL12-432 | 5´-CAT CTC AAG GTC ATT CCC-3´ | (3) |
| *T. medium* | S-S-Trep-I:B:C7-432 | 5´-CAT CAG ATG AGC ATT CCC-3´ | (3) |
| *T. phagedenis* | PT6 | 5'-CA TCA AGG ACG CAT TCC CTC-3' | (4) |
| *T. denticola* | S-S-Trep-T16-432 | 5´-CAT CTC ACA GGC ATT CCC-3´ | (3) |
| PT8 | S-S-Trep-DDK3-481 | 5´-CCC TTA TTC ACA TGA TTA CCG T-3´ | (1) |
| PT9 | PT9 | 5'-CT TCC TTT CCT TAC TAT CTC TTG-3' | (4) |
| *T. brennaborensis* | S-S-T.brenna-133 | 5´-CCT CAC AGC TCT CTA ACC TC-3´ | (5) |
| T. pedis | T. pedis | 5'-AG AGT CCT CAA CCT TTA CGT GTT-3' | (4) |
| PT12 | PT12-216 | 5'-CG AGC CCA TCT TTA GGC GAA G-3' | (4) |
| PT13 | PT13 | 5'-GT AGC TCC TTT CCC TTC ACC TTA A-3' | (4) |
| PT14 | Clon8-195 | 5'-CGG AGC TGA CGC TCC CTT-3' | (4) |
| PT15 | PT15 | 5'-CG TAG CTC CTT TCC ATA TAT GCT T-3' | (4) |
| *T. refringens* | Trep. refringens | 5'-GC TCC CTT TCC TTA CAT GAT-3' | (4) |
| PT18 | PT18 | 5'-CCGTCATCAGAGATGCATTC3' | (6) |
| *Dichelobacter nodosus* | S-S-D.nodosus-443 | 5'-CAT GCA CCG TTC TTC ACT-3' | (4) |

**Reference List**

1. **Choi BK, Nattermann H, Grund S, Haider W, Göbel UB.** 1997. Spirochetes from digital dermatitis lesions in cattle are closely related to treponemes associated with human periodontitis. Int. J. Syst. Bacteriol. **47**:175-181.
2. **Amann RI, Ludwig W, Schleifer KH.** 1995. Phylogenetic identification and in situ detection of individual microbial cells without cultivation. Microbiol. Rev. **59**:143-169.
3. **Klitgaard K, Boye M, Capion N, Jensen TK.** 2008. Evidence of multiple *Treponema* phylotypes involved in bovine digital dermatitis as shown by 16S rDNA analysis and fluorescent *in situ* hybridisation. J. Clin. Microbiol. **46**:3012-3020.
4. **Rasmussen M, Capion N, Klitgaard K, Rogdo T, Fjeldaas T, Boye M, Jensen TK.** 2012. Bovine digital dermatitis: Possible pathogenic consortium consisting of *Dichelobacter nodosus* and multiple *Treponema* species. Vet. Microbiol. **160**:151-161.
5. **Schrank K, Choi BK, Grund S, Moter A, Heuner K, Nattermann H, Göbel UB.** 1999. *Treponema brennaborense* sp. nov., a novel spirochaete isolated from a dairy cow suffering from digital dermatitis. Int. J. Syst. Bacteriol. **49**:43-50.
6. **Klitgaard Klitgaard K. Breto AF. Boye M, Jensen TK.** 2013. Targeting the Treponemal Microbiome of Digital Dermatitis Infections by High-Resolution Phylogenetic Analyses and Comparison with Fluorescent In Situ Hybridization, J. Clin. Microbiol. **51**: 2212-19.
